# Immunosuppressive property of submandibular lymph nodes in patients with head and neck tumors: differential distribution of regulatory T cells

**DOI:** 10.1186/s13104-018-3587-z

**Published:** 2018-07-16

**Authors:** Daiju Sakurai, Ryosuke Uchida, Fumie Ihara, Naoki Kunii, Takuya Nakagawa, Hideaki Chazono, Toyoyuki Hanazawa, Shinichiro Motohashi, Yoshitaka Okamoto

**Affiliations:** 1 Department of Otorhinolaryngology, Head and Neck Surgery, Graduate School of Medicine, Chiba University, 1-8-1 Inohana, Chuo-ku, Chiba, 260-8670 Japan; 2 Department of Medical Immunology, Graduate School of Medicine, Chiba University, 1-8-1 Inohana, Chuo-ku, Chiba, 260-8670 Japan

**Keywords:** Cervical lymph node, Dendritic cells, FcεRI, Head and neck tumor, Regulatory T cells, Submandibular lymph nodes, Upper jugular lymph nodes

## Abstract

**Objective:**

Different sensitizations and immune responses are thought to be induced in response to antigens at different mucosal sites between the oral floor and nose. The aim of this study was to investigate differences in the distributions of lymphocyte subsets in the submandibular (SM) and upper jugular (UJ) lymph nodes (LNs), which are supposed to be regional LNs of the oral floor and nasal mucosa, respectively. SMLNs and UJLNs were collected from patients with head and neck tumors who underwent surgical resection. The populations of T cells, Natural Killer (NK) cells, Natural Killer T (NKT) cells, regulatory T cells (Tregs) and dendritic cells (DCs) in LNs without metastasis were analyzed by flow cytometry. The high-affinity IgE receptor (FcεRI) expression of LN cells were also evaluated.

**Results:**

The proportions of CD4^+^CD25^+^Foxp3^+^ Tregs, CD4^+^CD45RA^−^Foxp3^high^ effector Tregs and FcεRIα^+^CD33^+^CD11c^+^ DCs were significantly larger in SMLNs compared with UJLNs, while those of CD3^+^ T cells, CD3^−^CD56^+^ NK cells, CD3^+^Vα24^+^Vβ11^+^ NKT cells, and CD123^+^CD303^+^ DCs did not show any significant differences between SMLNs and UJLNs. The differential distributions of CD4^+^CD25^+^Foxp3^+^ Tregs were observed regardless of tumor region, LN metastasis and clinical staging. These data indicate that SMLNs may have immunosuppressive properties compared with UJLNs.

**Electronic supplementary material:**

The online version of this article (10.1186/s13104-018-3587-z) contains supplementary material, which is available to authorized users.

## Introduction

The nasal mucosa and oral mucosa are located at the entrance of the respiratory tract and gastrointestinal tract, respectively, and are constantly exposed to various antigens. However, different sensitizations and immune responses are induced in response to antigens at these different mucosal sites. When the nasal mucosa is exposed to allergens, specific IgE production is evoked, and following repeated exposure to the allergen, typical nasal symptoms of allergic rhinitis (AR) are induced. In contrast, the oral cavity is exposed to various foreign substances, such as foods, bacteria, and viruses, but excessive immune responses, such as oral allergy syndrome, are normally restrained. Sublingual immunotherapy (SLIT), in which an allergen is applied to the oral floor in patients with various allergic diseases, attenuates allergic reactions [1, 2]. Conversely, the administration of a viral vaccine, such as the influenza vaccine, to the nasal mucosa enhances immune responses against the virus [3, 4]. It is therefore known that there are differential immune responses between the nasal mucosa and oral mucosa following antigen exposure.

Dendritic cells (DCs) capture antigens exposed to the mucosa and migrate to regional lymph nodes (LNs), where they present the antigen to lymphocytes [5]. Differential characteristics of DCs between the oral and nasal mucosa have been previously reported [6]. The different immunocompetent cells at various mucosal sites are expected to induce different immune responses.

It has also been reported that cultured DCs administered to the nasal submucosa quickly migrate to upper jugular lymph nodes (UJLNs), while those administered to the oral floor mucosa migrate to submandibular lymph nodes (SMLNs) [8, 9]. A significant increase in the number of peripheral Natural Killer T cells (NKT cells) has been observed following the nasal submucosal administration of DCs pulsed with α-galactosylceramide (αGalCer), a specific ligand for invariant NKT cells, while this response was not observed following administration in the oral floor mucosa [8]. These results suggest that the differential immunological responses observed between the nasal and oral mucosa depend upon differences in each type of mucosa, including sites involving DCs as well as differences in draining LNs.

In this study, we investigated the differential distribution of lymphocyte subsets between SMLNs and UJLNs collected from the surgical specimens of patients with head and neck tumors.

## Main text

### Materials and methods

#### Patient samples

30 patients between 36 and 84 years old with head and neck tumors were enrolled in this study (Additional file 1: Table S1). All patients underwent surgery in the Department of Otorhinolaryngology and Head and Neck Surgery, Chiba University Hospital. We sorted the dissected LNs into different LN regions (SMLNs and UJLNs) immediately after the operation. A representative LN ranging from 7 to 10 mm in diameter from SMLN and UJLN regions of each patient was collected, and split samples of approximately 5 mm^3^ from these lymph nodes were used for analysis. Approximately 1 × 10^7^ cells were collected from the segments, and other segments were submitted for pathological diagnosis. LNs without metastasis were confirmed by pathological examination. The study was approved by the Ethics Committee of Chiba University. Written informed consent was obtained from each patient prior to participation in the study.

#### Lymph node mononuclear cells (LNMCs)

The collected LNs were placed in complete RPMI1640 medium, and then homogenized to generate LNMCs, which were filtered through a nylon mesh and washed twice in complete RPMI1640 medium.

#### Flow cytometry

LNMCs were stained with the following anti-human antibodies: anti-CD3 APC, anti-CD56 PE-Cy7, anti-CD4 PacificBlue, anti-CD25 PE, anti-CD45RA FITC, anti-CD33 FITC (BD Bioscience, Franklin Lakes, NJ, USA), anti-Vα24 FITC, anti-Vβ11 (Beckman Coulter, Brea, CA, USA), anti-CD123 eFluor450, anti-FcεRIα PE (Affymetrix, Santa Clara, CA, USA), and anti-CD303 FITC (Miltenyi Biotec, Bergisch Gladbach, Germany) for surface staining. Anti-Foxp3-eflour660 (eBioscience) was used for intracellular staining. The Fixation/Permeabilization Diluent and Concentrate, and Permeabilization Buffer (all from Affymetrix) were used to fix and permeabilized the cells according to the manufacturer’s instructions. Cellular phenotypes were evaluated using the FACSCanto II system (BD Biosciences). Data were analyzed using FlowJo software (Tree Star Inc., Ashland, OR, USA).

#### Statistical analysis

Statistical analyses were performed using the paired and unpaired *t* test. A value of p < 0.05 was considered statistically significant.

### Results

#### Differential distribution of CD4^+^CD25^+^Foxp3^+^ regulatory T cells (Tregs) between SMLNs and UJLNs

**Fig. 1:**
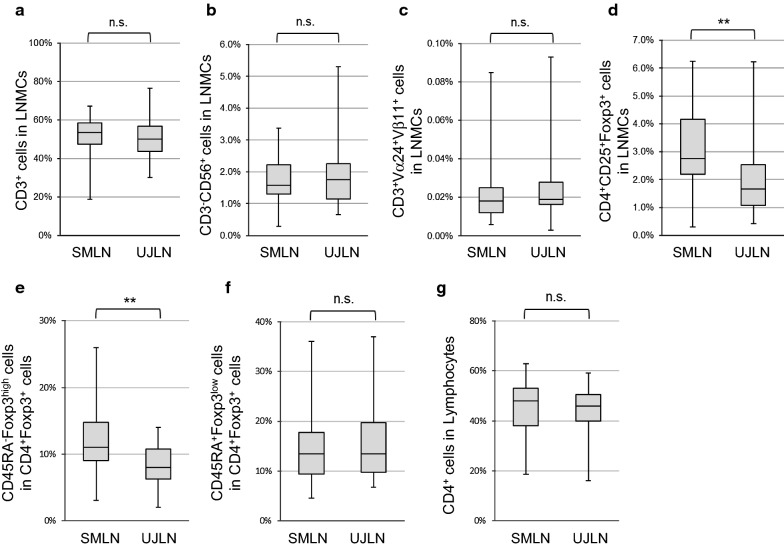
The distributions of lymphocyte subsets in SMLNs and UJLNs. The proportions of CD3^+^ cells (**a**), CD3^−^CD56^+^ cells (**b**), CD3^+^Vα24^+^Vβ11^+^ cells (**c**), CD4^+^CD25^+^Foxp3^+^ cells (**d**), CD4^+^CD45RA^−^Foxp3^high^ cells (**e**), CD4^+^CD45RA^+^Foxp3^low^ cells (**f**), and CD4^+^ cells (**g**) among the LNMCs of SMLNs and UJLNs are shown (n = 30). The data are shown as boxplots. The central rectangle spans indicate the first quartile to the third quartile. The segment inside the rectangle shows the median and whiskers above and below the box show minimum and maximum values, respectively. The p values were obtained by the unpaired t-test. **p < 0.01; *n.s.* not significant

To investigate the characteristics of LNMCs extracted from SMLNs and UJLNs, the proportions of CD3^+^ T cells, CD3^−^CD56^+^ NK cells, CD3^+^Vα24^+^Vβ11^+^ NKT cells and CD4^+^CD25^+^Foxp3^+^ Tregs in the LNMCs were analyzed. The populations of CD3^+^ cells, CD3^−^CD56^+^ cells and CD3^+^Vα24^+^Vβ11^+^ cells in SMLNs and UJLNs did not show any significant differences (Fig. 1a–c). The proportion of CD4^+^CD25^+^Foxp3^+^ cells in SMLNs was significantly larger than in UJLNs (p < 0.01; Fig. 1d). The subsets of CD4^+^Foxp3^+^ Tregs were analyzed based on CD45RA and Foxp3 expression. The proportion of CD45RA^−^Foxp3^high^ cells among CD4^+^Foxp3^+^ cells in SMLNs was significantly larger than in UJLNs (p < 0.01; Fig. 1e). Conversely, the proportion of CD45RA^+^Foxp3^low^ cells among the CD4^+^Foxp3^+^ cells in SMLNs and UJLNs were not significantly different (Fig. 1f). The proportion of CD4^+^ T cells among the LNMCs were not significantly different (Fig. 1g).

#### Increased CD4^+^CD25^+^Foxp3^+^ Tregs in SMLNs independent of clinical features

**Fig. 2:**
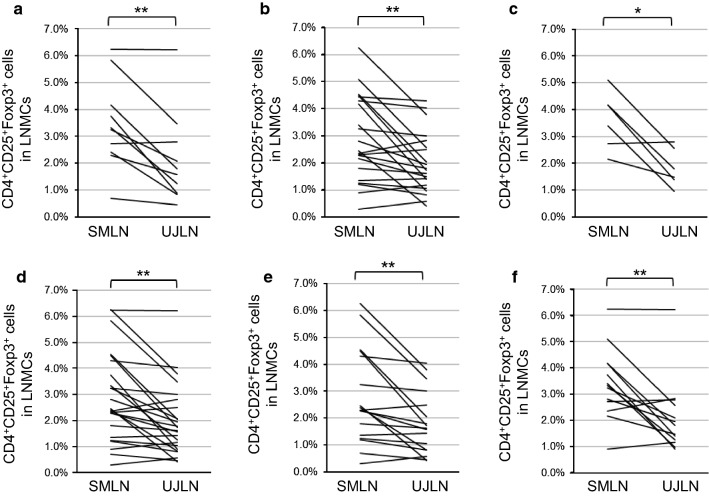
The proportion of CD4^+^CD25^+^Foxp3^+^ Tregs among LNMCs from SMLNs and UJLNs based on clinical features. The percentage of CD4^+^CD25^+^Foxp3^+^ cells among LNMCs from SMLNs and UJLNs are shown (**a**–**f**). The tongue and oral cavity tumor patients are shown in (**a**) (n = 10), and those with tumors in other regions are shown in (**b**) (n = 20). An early-stage group, which included patients with stage I, II and benign tumors (n = 6) (**c**), and an advanced-stage group, which included stage III, IV and recurrent tumors (n = 24) (**d**). The results of an analysis of patients with LN metastasis are shown in (**e**) (n = 17), while those of patients without LN metastasis are shown in (**f**) (n = 13). The p-values were obtained by the paired t-test. *p < 0.05; **p < 0.01; *n.s.* not significant

The proportions of CD4^+^CD25^+^Foxp3^+^ cells among LNMCs from SMLNs were significantly larger than those of UJLNs collected from patients with both oral cancer (p < 0.01; Fig. 2a) and cancer at other sites (p < 0.01; Fig. 2b), in both early- (p < 0.05; Fig. 2c) and advanced-stage (p < 0.01; Fig. 2d) cases, and in patients with or without LN metastasis (p < 0.01 and p < 0.01, respectively; Fig. 2e, f).

#### Increased proportion of FcεRI^+^CD33^+^CD11c^+^ cells in SMLNs

**Fig. 3:**
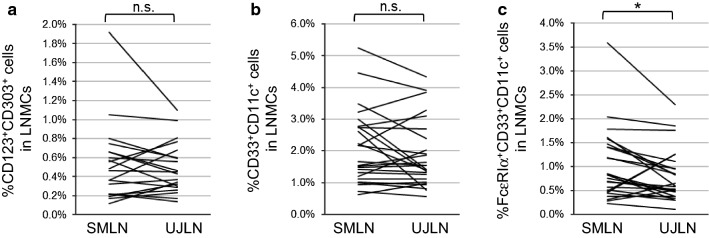
The distribution of CD123^+^CD303^+^ cells and CD11c^+^CD33^+^ cells among LNMCs from SMLNs and UJLNs. The proportions of CD123^+^CD303^+^ cells (**a**), CD11c^+^CD33^+^ cells (**b**) and FcεRIα^+^CD11c^+^CD33^+^ cells (**c**) among LNMCs from SMLNs and UJLNs are shown. The p-values were obtained by the paired t-test. *p < 0.05; *n.s.* not significant

The proportions of both CD123^+^CD303^+^ and CD33^+^CD11c^+^ cells in the LNMCs did not show any significant differences between SMLNs and UJLNs (Fig. 3a, b). However, the proportion of FcεRIα^+^CD33^+^CD11c^+^ cells in LNMCs was significantly larger in SMLNs than UJLNs (p < 0.05; Fig. 3c).

### Discussion

In this study, the immunological differences of LNMCs between SMLNs and UJLNs were investigated, and our data suggested that SMLNs had more immunosuppressive properties than UJLNs. We first examined the proportion of lymphocytes between SMLNs and UJLNs, but no significant differences in CD3^+^ T cells, CD3^−^CD56^+^ NK cells or CD3^+^Vα24^+^Vβ11^+^ NKT cells were observed. However, significantly higher proportions of CD4^+^CD25^+^Foxp3^+^ Tregs were detected in SMLNs than in UJLNs. Recently, CD4^+^Foxp3^+^ Tregs were reported to be further subdivided into functionally distinct subpopulations based on CD45RA and Foxp3 expression [10]. CD45RA^−^Foxp3^high^ Tregs expressed more CTLA-4 and IL-2 (CD25) receptor on their cell surfaces and had potent immunosuppressive activities for T cells, and were thus called effector Tregs [11, 12]. In this study, a significantly higher proportion of CD4^+^CD45RA^−^Foxp3^high^ effector Tregs was found in SMLNs compared with UJLNs.

The LNMCs from the SMLNs and UJLNs used in this study were collected from surgically resected specimens from patients with head and neck tumors. The frequency of Tregs in the peripheral blood has been reported to be elevated in patients with cancer, including those with head and neck cancer [13, 14]. These results might reflect the increased regulatory T cells in LNs with metastasis and primary tumors in cancer patients which were associated with clinical stage and the presence of lymph node metastasis [13]. Therefore, we examined the influence of tumor-related factors such as the primary tumor region, the presence of cervical LN metastasis and clinical stage on differences in the Treg proportions between SMLNs and UJLNs. However, significant differences in the proportion of CD4^+^CD25^+^Foxp3^+^ Tregs between SMLNs and UJLNs remained, regardless of the clinical features examined. This suggested that increased Tregs were characteristic of SMLNs compared with UJLNs.

In a previous study, DCs administered to the nasal mucosa migrated to UJLNs, while those administered to the oral floor mucosa migrated to SMLNs [8, 9]. Additionally, a significant increase in the number of peripheral NKT cells has been observed after administering DCs pulsed with the NKT cell ligand αGalCer into the nasal mucosa. However, these activities were not detected after administering DCs into the oral floor mucosa [8]. Although the mechanisms remain unknown, it has been suggested that the NKT cell activation by DCs that migrated from the oral floor mucosa might be inhibited by the increased Tregs in SMLNs, which are the draining LNs of the oral floor mucosa.

The induction and expansion of Tregs have been shown to be controlled by DCs [15, 16]. Human DCs have two major subtypes, conventional DCs (cDCs) and plasmacytoid DCs (pDCs) [17]. cDCs have can stimulate T cells, evoking Th1- or Th2-responses, depending on the inflammatory environment [18–20]. pDCs produce type I interferon when there is an infection [18], and it has been suggested that they induce T cell tolerance [18, 20]. In this study, although we analyzed CD123^+^CD303^+^ pDCs and CD33^+^CD11c^+^ cells which included cDC, macrophage and monocyte populations, no differences were found between SMLNs and UJLNs.

The oral mucosa is known to have a lot of DCs that express FcεRI [7, 21], and allergens can be taken up by IgE molecules bound to the FcεRI expressed on oral DCs [22]. In this study, a larger proportion of FcεRI-expressing CD33^+^CD11c^+^ cells was observed in submandibular LNMCs compared with upper jugular LNMCs. These FcεRI-expressing cDCs might migrate to the SMLNs from the oral mucosa, where they induce Treg and immune tolerance to various commonly-exposed antigens or to allergens administrated by sublingual immunotherapy [7, 23].

## Conclusion

In this study, we identified a differential distribution of lymphocyte subsets between SMLNs and UJLNs. The proportions of CD4^+^CD25^+^Foxp3^+^ Tregs, CD4^+^CD45RA^−^Foxp3^high^ effector Tregs and FcεRI-expressing CD33^+^CD11c^+^ cells in SMLNs were larger than those in UJLNs. SMLNs may have more potent immunosuppressive properties than UJLNs.

## Limitations

In this study, we examined the difference in the distribution of immune cells in two LN regions. However, a limitation of this study is that the samples were restricted to tumor patients who underwent surgery because it is unethical to collect LNs from healthy subjects. It is possible that the distribution of immune cells in LNs differed between patients with head and neck tumors and healthy subjects, but it is difficult to compare immune cell profiles between these groups. Additionally, the detailed interactions between Tregs and FcεRI-expressing CD33^+^CD11c^+^ cells, and their suppressive functions and effects on immune cells in lymph nodes needs further investigation.

## Additional file

**Additional file 1: Table S1.** Clinical characteristics of the head and neck tumor patients. Clinical characteristics of the head and neck tumor patients enrolled in this study.

## Abbreviations

αGalCer: α-galactosylceramide
AR: allergic rhinitis
cDC: conventional dendritic cell
DC: dendritic cell
LNMC: lymph node mononuclear cell
NK cell: Natural Killer cell
NKT cell: Natural Killer T cell
pDC: plasmacytoid dendritic cell
SLIT: sublingual immunotherapy
SMLN: submandibular lymph node
Treg: regulatory T cell
FcεRI: the high-affinity IgE receptor
UJLN: upper jugular lymph node
