# Supplementary material for: Immunosuppressive property of submandibular lymph nodes in patients with head and neck tumors: differential distribution of regulatory T cells
Source: BMC Res Notes. 2018 Jul 16;11:479. doi: 10.1186/s13104-018-3587-z (PMC6048882; doi:10.1186/s13104-018-3587-z)
Supplement: Supplementary file 1 — Additional file 1: Table S1. Clinical characteristics of the head and neck tumor patients. Clinical characteristics of the head and neck tumor patients enrolled in this study. [file 13104_2018_3587_MOESM1_ESM.docx]

|  | Tumor lesion | Pathology | Age/gender | Stage | Past treatment |
| --- | --- | --- | --- | --- | --- |
| 1 | Tongue | SCC | 64/M | II |  |
| 2 | Hypopharynx | SCC | 72/M | IV |  |
| 3 | Larynx | SCC | 75/M | IV | RT |
| 4 | Parotid gland | SCC | 69/M | I |  |
| 5 | Tongue | SCC | 75/F | II |  |
| 6 | Oral cavity | SCC | 71/M | III |  |
| 7 | Tongue | SCC | 73/M | recurrence | Ope |
| 8 | Maxillary sinus | SCC | 75/M | IV |  |
| 9 | Oral cavity | SCC | 73/M | IV | CRT |
| 10 | Parotid gland | Mucoepidermoid carcinoma | 75/F | II |  |
| 11 | Tongue | SCC | 39/M | III |  |
| 12 | Thyroid | Papillary adenocarcinoma | 66/M | III |  |
| 13 | Maxillary sinus | SCC | 51/M | recurrence | RT |
| 14 | Thyroid | Papillary adenocarcinoma | 67/F | III |  |
| 15 | Carotid　body tumor | Benign | 72/F | − |  |
| 16 | Parotid gland | Acinic cell carcinoma | 36/M | III |  |
| 17 | Hypopharynx | SCC | 68/M | recurrence | CRT, Ope |
| 18 | Larynx | SCC | 84/M | III |  |
| 19 | Larynx | SCC | 68/M | IV |  |
| 20 | Parotid gland | adenoid cytic carcinoma | 70/M | IV |  |
| 21 | Tongue | SCC | 74/M | IV |  |
| 22 | Parotid gland | adeno carcinoma | 62/M | II |  |
| 23 | Tongue | SCC | 71/M | IV |  |
| 24 | Hypopharynx | Adenoid cystic carcinoma | 61/F | III |  |
| 25 | Tongue | SCC | 64/M | III |  |
| 26 | Oropharynx | SCC | 61/M | IV | CRT |
| 27 | Hypopharynx | SCC | 63/M | IV |  |
| 28 | Oral cavity | SCC | 53/M | IV |  |
| 29 | Maxillary sinus | Malignant melanoma | 76/M | III |  |
| 30 | Parotid gland | Salivary duct carcinoma | 46/M | IV |  |

Table S1. Clinical characteristics of the head and neck tumor patients
